# Usefulness and limitations of dK random graph models to predict interactions and functional homogeneity in biological networks under a pseudo-likelihood parameter estimation approach

**DOI:** 10.1186/1471-2105-10-277

**Published:** 2009-09-03

**Authors:** Wenhui Wang, Juan Nunez-Iglesias, Yihui Luan, Fengzhu Sun

**Affiliations:** 1 School of Mathematics, Shandong University, Jinan, Shandong 250100, PR China; 2 Molecular and Computational Biology Program, University of Southern California, Los Angeles, CA 90089-2910, USA; 3 MOE Key Laboratory of Bioinformatics and Bioinformatics Division, TNLIST/Department of Automation, Tsinghua University, Beijing, PR China

## Abstract

**Background:**

Many aspects of biological functions can be modeled by biological networks, such as protein interaction networks, metabolic networks, and gene coexpression networks. Studying the statistical properties of these networks in turn allows us to infer biological function. Complex statistical network models can potentially more accurately describe the networks, but it is not clear whether such complex models are better suited to find biologically meaningful subnetworks.

**Results:**

Recent studies have shown that the degree distribution of the nodes is not an adequate statistic in many molecular networks. We sought to extend this statistic with 2nd and 3rd order degree correlations and developed a pseudo-likelihood approach to estimate the parameters. The approach was used to analyze the MIPS and BIOGRID yeast protein interaction networks, and two yeast coexpression networks. We showed that 2nd order degree correlation information gave better predictions of gene interactions in both protein interaction and gene coexpression networks. However, in the biologically important task of predicting functionally homogeneous modules, degree correlation information performs marginally better in the case of the MIPS and BIOGRID protein interaction networks, but worse in the case of gene coexpression networks.

**Conclusion:**

Our use of dK models showed that incorporation of degree correlations could increase predictive power in some contexts, albeit sometimes marginally, but, in all contexts, the use of third-order degree correlations decreased accuracy. However, it is possible that other parameter estimation methods, such as maximum likelihood, will show the usefulness of incorporating 2nd and 3rd degree correlations in predicting functionally homogeneous modules.

## Background

High throughput technologies such as microarrays and yeast-two-hybrid assays have resulted in an explosion of biological data that can be represented as networks. For example, microarray datasets can be analyzed as a *coexpression network*, in which nodes (or vertices) represent genes and links (or edges) represent coexpression, the similarity of the level of expression of two genes over the samples in the study. Similarly, protein interaction data, such as that generated by yeast-two-hybrid assays, can be summarized as a network, with nodes representing proteins and edges representing physical interaction between two proteins.

Genes and their products give rise to biological function through their interaction with each other and with other components of the cell. The analysis of the above biological networks is therefore the natural way to understand cellular function on a genome-wide level. In particular, we need a thorough understanding of the statistical properties of biological networks if we aim to make inferences, such as inferring evolutionary relationships between various networks, or separating signal from noise in imperfect network data.

Erdős and Rényi [1] were the first to study the statistical properties of random graph models. In their models (now known as ER models), any edge between two vertices occurred independently of other edges with a constant probability *p*. In these graphs, however, the degree of a vertex (the number of links to other vertices) is a random variable with an approximately Poisson distribution with *λ *= (*n *- 1)*p*, which is grossly at odds with most biological network observed to date [2,3].

In real biological data, node degrees usually have heavy tail distributions [2,3]. Accordingly, in most statistical studies of biological networks, the null model is a random graph from the set having a degree distribution identical to that of the data, or a distribution in which the expected degrees are identical to those observed in the data [4].

These models are themselves limited, because in addition to their degree distributions, biological networks show highly clustered connections [5] and transitivity [6]. Indeed, it is difficult to assess which properties of a network would represent sufficient statistics that are biologically meaningful.

Mahadevan et al. [7] attempted to solve this problem by devising an increasing series of random network models they referred as the *dK*-series. The distributions of the random networks are defined as uniform over the set of graphs having the same distribution of *d*-sized subgraphs as the observed network data. Particular cases of the series reduce to familiar distributions: the 0*K *distribution *P*_0 _is identical to the corresponding ER distribution, which describes the average number of links per node. The 1*K *distribution model tells us the expected degree of each node and assumes that the nodes are randomly connected conditional on the expected node degrees. The 2*K *distribution *P*_2 _describes the interconnectivity of nodes with given degrees, maintaining the number (*m*(*k, k'*)) of links between nodes of degrees *k *and *k'*. The 2*K *distribution therefore preserves degree-degree correlations between nodes (known as the assortativity of the network). Including still more connectivity information, the 3*K *distribution considers degree correlations among any 3 nodes, which include the transitivity of the network. Moving beyond pairs of nodes, various topological structures are possible. For example, there are 8 different kinds of isomorphic structures for the 3*K *distribution. Increasingly larger subgraphs can be enumerated for *d *= 4,5,..., capturing increasingly complex features of a particular graph.

The *dK*-series is therefore an objective way to progressively include more features into a random graph model, just as each term in a Fourier or Taylor series progressively captures more details of a given function, and thus largely avoids the arbitrary selection of statistics that may or may not be sufficient or relevant to a particular process.

Using this series as our starting point, we sought to evaluate the use of ever more inclusive dK distributions in the study of biological networks. For four different biological networks, we trained *dK *models for *d *= 0, 1, 2, 3. We first explored the properties of the models by evaluating their ability to predict in the observed networks the presence or absence of individual edges, as well as general network statistics. We showed that the 2K model outperforms other models in predicting the presence and absence of the edges for both protein interaction and gene coexpression networks.

We then evaluated whether statistical significance against one of the models for subnetworks corresponded to biological significance. We modeled our approach based on the scoring scheme used by Tanay et al. [8,9]: they devised a pseudo-likelihood score for edges in a bipartite graph of genes and samples, in which edges occur according to a null model that corresponds to the 1*K *distribution, or to an alternative model, representing biological significance, independently with a high constant probability *p *= 0.9. They showed that this score results in improved accuracy in predicting functional gene groups, when compared with network density alone (which is equivalent to using 0*K *as the null model).

We reproduced their score using as the null distribution one of *dK *models for *d *= 0, 1, 2, 3. We aimed to test the hypothesis that more inclusive distributions would result in a score for a set of nodes that is more indicative of biological significance, just as 1*K *was in the case of bipartite graphs. We were surprised, however, to find that accuracy was only slightly increased with each successive *dK *distribution in the case of yeast protein interaction networks, while the 0*K *distribution (equivalent to edge density) had the best predictive power in coexpression networks.

## Results and Discussion

In this section, we first give a brief discussion of the *dK *models and the pseudo-likelihood methods for estimating the parameters. Next we study the accuracy of the *dK *models in predicting edges in the molecular networks. Then several statistics related to the networks are studied to evaluate if the random networks can approximate the observed networks. Finally, we evaluate if the *dK *models can be used to identify functionally homogeneous modules.

### Model description and parameter estimation

For each network, we created random graph models matching the 0*K*, 1*K*, 2*K*, and 3*K *distributions of the observed network. For the 0K and 1K models, we took the degree sequence and number of edges as fixed properties of the network and thus defined the matching models. In the 0K model, each edge occurs independently with probability , |*E*| being the number of edges and |*V*| the number of nodes in the real network. In the 1K model, each edge occurs independently, conditional on the degrees *k*_1 _and *k*_2 _of its incident nodes, with probability *p*(*k*_1_, *k*_2_) = min (*k*_1_*k*_2_/(2·|*E*|), 1 - *ε*), for *ε *small (in our case, 10^-4^). For the 2K model, we calculated the probability *p*(*k*_1_, *k*_2_) that two proteins with degree pairs (*k*_1_, *k*_2_) interact. One intuitive approach is to estimate *p*(*k*_1_, *k*_2_) by the fraction of interacting protein pairs among all the protein pairs with degrees (*k*_1_, *k*_2_), *k*_1 _≤ *k*_2_. However, for many degree pairs (*k*_1_, *k*_2_), the number of such protein pairs is small. Thus, the estimated value of *p*(*k*_1_, *k*_2_) using this intuitive approach is not reliable. To overcome this problem, we modeled *p*(*k*_1_, *k*_2_) as a function of (*k*_1_, *k*_2_) and fitted the function using Matlab. Details of the estimation method are given in the "Methods" section.

**Figure 1:**
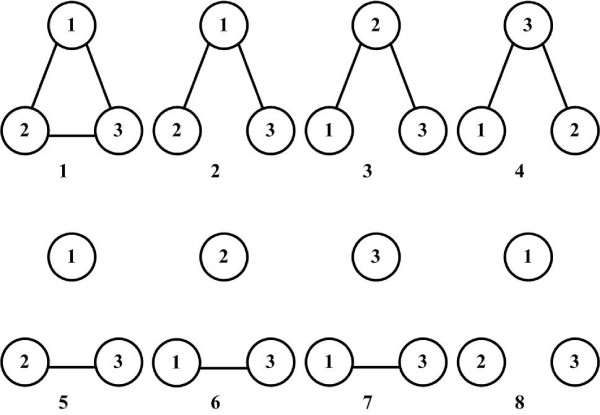
**Possible different triplet topologies**. Classification of triplets of nodes into eight different categories based on their connectivity and degrees.

The 3K model describes how protein triplets with degrees (*k*_1_, *k*_2_, *k*_3_) interact with each other. There are a total of eight possible interaction patterns among the three proteins as shown in Figure 1. As in the 2K model, directly estimating the eight probabilities corresponding to the interacting patterns is difficult due to the small number of protein triplets for many degree triplets (*k*_1_, *k*_2_, *k*_3_). Thus, we reparameterized the probabilities to fit a logistic regression model, which is necessary to improve probability estimates for degree triplets for which we only have one or few examples. Details are given in the "Methods" section.

### The performance of predicting protein interactions using the *dK *distribution models

**Figure 2:**
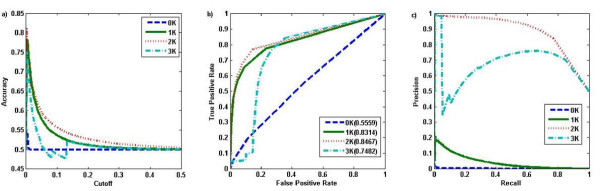
**The performance of the *dK *distribution models in predicting protein interactions for the MIPS interaction data**. a) The prediction accuracy versus the cut-off threshold for the interaction probability; b) ROC curve; c) Precision-Recall.

**Figure 3:**
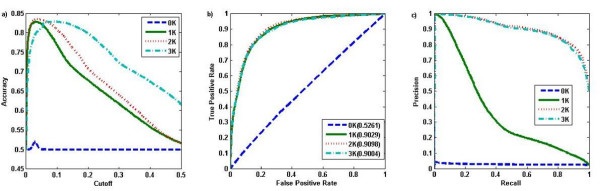
**The performance of the *dK *distribution models in predicting protein interactions for the GDS1013 expression data (PCC cut-off threshold 0.89)**. a) The prediction accuracy versus the cut-off threshold for the interaction probability; b) ROC curve; c) Precision-Recall.

We began by studying the ability of the *dK *distribution models in their capacity to predict the presence and absence of links in the observed biological networks. For each *dK *(*d *= 0, 1, 2, 3) distribution model, we predicted the probability that any pair of proteins interact using the estimated parameters obtained above. Given a cut-off threshold, protein pairs with interaction probability above the threshold were predicted to interact. The predicted interaction pairs were compared with the observed protein interactions to study the ability of the *dK *distribution model to predict protein interactions. To overcome the problem of extreme large number of non-interacting protein pairs over that of the interacting pairs, we randomly chose the same number of non-interacting protein pairs as the number of interacting pairs for the comparisons. Three different evaluation methods were used: the accuracy, the receiver operation curve (ROC), and the precision-recall curve. Figure 2 and Figure 3 show the performance of the *dK *models in predicting protein interactions using the MIPS [10] protein interaction data and a gene co-expression network based on GDS1013 [11], respectively. The performance of the 2*K *distribution model always outperformed the 1*K *distribution model, which in turn outperformed the 0*K *distribution. The 3*K *distribution model performed comparably to the 2*K *distribution model in a coexpression network (Figure 3), but significantly worse in the case of a protein interaction network (Figure 2). Similar results were obtained for the BIOGRID [12] protein interaction data (Additional file 1: Figure S1) and other coexpression networks (Additional file 1: Figures S2-S4).

### Comparing statistical features of random networks from the *dK *models with that of the observed networks

We next studied if the random networks based on the *dK *distribution models approximate the observed interaction networks. To achieve this objective, we generated 100 random networks based on the *dK *distribution models and calculated several statistical features of the random networks (see "Methods" for details). We studied five network statistical features as in [7]:

• *λ*_1_: average of the smallest eigenvalue of the Laplacian of the graph matrix;

• *λ*_*n*-1_: average of the largest eigenvalue of the Laplacian of the graph matrix;

• *d*: average shortest distance between the nodes;

• *σ*_*d*_: standard deviation of shortest distance between the nodes;

• *r*: average assortativity coefficients.

The elements of the Laplacian matrix of a network are defined by  if node *i *with degree *k*_*i *_and node *j *with degree *k*_*j *_are connected and *l*_*ij *_= 0 otherwise for *i *≠ *j*, and *l*_*ij *_= 1 if *i *= *j*. Several other important network statistics [7], e.g. network resilience and performance, are tightly controlled by the smallest non-zero (*λ*_1_) and the largest (*λ*_*n*-1_) eigenvalues of the Laplacian matrix. Therefore, we studied whether the corresponding eigenvalues of the dK random networks are close to that of the true network. In addition, the distribution of the shortest distances between any two nodes provides information on how the nodes cluster together in the network. We used two quantities, the mean and standard deviation of the shortest distances, to characterize this distribution. Finally, the assortativity coefficient of a network provides information on how nodes of different degrees link to each other. Although these five network statistical measures cannot fully describe the network of interest, they capture important network properties. If the dK distribution models can approximate the true network well, these quantities in the dK random networks should be close to the corresponding values of the true network.

Tables 1 and 2 give the average and standard deviation of the corresponding feature values from the 100 random networks for each of the *dK *distribution models based on the MIPS protein interaction network and GDS1013 coexpression network, respectively. For the MIPS protein interaction data, the average values of the five network features for the random networks converge to the corresponding values of the observed network, indicating that the *dK *model converges to the true network as *d *increases. On the other hand, for the coexpression network, the average values of the five network features of the 2*K *model are closer to the corresponding features in the true network than the 1*K *and 3*K *models. The poor performance of the 3*K *model maybe due to the fact that the estimated parameters are not accurate due to the relative small number of the nodes in the coexpression networks. Similar tables for the other networks are provided in Additional file 2: Tables S1-S4.

**Table 1:** Comparison of five network features for the *dK *distribution models with that of the MIPS protein interaction network, *d *= 1, 2, 3.


**Metric**	***λ***_**1**_	***λ***_***n*-1**_	***d***	***σ***_***d***_	***r***

MIPS	0.03	1.97	4.42	1.12	-0.14

1k	0.07(0.018)	1.93(0.018)	3.95(0.0117)	0.9045	-0.07(0.0058)

2k	0.06(0.014)	1.94(0.014)	4.04(0.0097)	0.9679	-0.12(0.0035)

3k	0.04(0.014)	1.96(0.014)	4.26(0.0107)	1.0613	-0.14(0.0014)

*λ*_1_: average of the smallest eigenvalue of the Laplacian of the graph matrix; *λ*_*n*-1_: average of the largest eigenvalue of the Laplacian of the graph matrix; *d*: average shortest distance between the nodes; *σ*_*d*_: standard deviation of shortest distance between the nodes; *r*: average assortativity coefficients. The quantity in the brackets indicate the standard deviation of corresponding metric

**Table 2:** Comparison of five network features for the *dK *distribution models with that of the GDS1013 coexpression network with PCC cut-off threshold of 0.89, *d *= 1, 2, 3.


**Metric**	***λ***_**1**_	***λ***_***n*-1**_	***d***	***σ***_***d***_	***r***

Coexp	0.09	1.91	3.27	1.31	0.5

1k	0.31(0.068)	1.69(0.068)	2.61(0.0053)	0.67	-0.05(0.0052)

2k	0.12(0.038)	1.88(0.038)	2.78(0.0013)	0.83	0.20(0.0037)

3k	0.19(0.059)	1.81(0.059)	2.69(0.0074)	0.74	0.12(0.0042)

Notations are the same as in Table 1.

### The performance of the *dK *distribution models for the identification of functionally homogeneous modules

Our primary motivation of this study is to see if the more complex models, which can generally more accurately describe the observed network, are helpful in the identification of biologically functionally homogeneous modules. Statistical deviations from a suitable model would indicate evolutionary pressure and thus functional significance. Therefore, we can compare the functional relevance of each model by how well statistical deviations from the model correlate with the functional homogeneity of the corresponding nodes.

We designed scores from our models based on an alternative hypothesis that edges are present in a functional module with constant probability *p*. Generally *p *should be close to 1 as most functionally homogeneous modules are highly clustered. As in [8,9], we chose *p *= 0.9 in the main text. To see the validity of our results for different values of *p*, we also changed *p *to *p *= 0.85 and *p *= 0.95. Our approach is similar to that used by Tanay *et al *[8,9], which they used a single null model (a graph is chosen at random from the set of networks having identical degree sequence to the original network, equivalent to our 1K model) in the context of a bipartite graph. With this score framework, we used a simulated annealing algorithm to find groups of genes with high scores, retaining every group encountered during the run of the algorithm and their scores under each of the null models.

Finally, we called a gene group *functionally homogeneous *if it was enriched in at least one functional category from the Gene Ontology [13]. We defined module enrichment by the hypergeometric test *p*-value, with a threshold of *p *< 10^-5^. These gene groups were taken to be true positives, and the remaining gene groups were taken to be true negatives. Again, we varied this threshold from 0.01 to 10^-6^, and no qualitative changes in the results were observed, showing that our approach is robust to the parameter for calling functional homogeneity (data not shown). We then evaluated the four models by comparing how well they can predict functional homogeneity in the MIPS and BIOGRID yeast protein interaction networks, or in two different yeast gene coexpression networks, GDS1013 and GDS1103.

#### Results based on the MIPS protein interaction network

**Figure 4:**
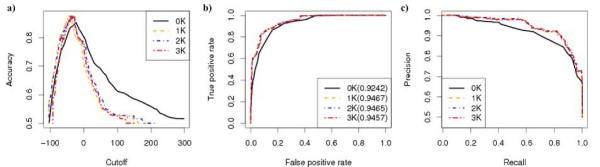
**The performance of *dK *distribution models in predicting functionally homogeneous modules based on MIPS interaction data**. The gene group size was 10, the p-value cut-off was 10^-5^, and *p *= 0.9. a) Accuracy; b) ROC curve; c) Precision-Recall

We used the MIPS [10] yeast protein interaction network to compare the ability of the *dK *distributions to predict functional homogeneity in gene groups of size 10. Note that the size of the gene groups cannot be too small. Otherwise it is very hard to distinguish functionally homogeneous modules from random gene groups. The score for each gene group depends on the null model for the network. We calculated the score for gene groups of size 10 using dK distribution model as the null network model, *d *= 0, 1, 2, 3. A gene set was predicted to be functional homogeneous if the score is above a cut-off threshold. Our objective is to see which score functions can more accurately predict functional homogeneity. Therefore, we compare the predicted "functionally homogeneous" gene groups with the positive groups. We measure the performance using accuracy, the ROC curve, and the precision-recall curve as above. Note that the subnetwork scores defined based on *d*K models in equation (7) for *d *= 0, 1, 2 and equation (9) for *d *= 3 are not on the same scale and thus the prediction accuracy in Figure 4a is not comparable for the same cut-off value of the subnetwork scores. However, the maximum prediction accuracy for the dK models can be compared. Figure 4 shows the results based on the MIPS interaction data with *p *= 0.9 and gene group size 10. The results based on other combinations of *p *= 0.85, 0.9, 0.95 and gene set size *n *= 8, 10 are given in Additional file 1: Figures S5-S7. The corresponding results based on the BIOGRID [12] protein interaction data are also given as Additional file 1: Figures S8-S11.

We found almost no difference between the performance of the different scores, with even the 0K model (density) performing only slightly worse than the rest. Figure 4a shows that the highest prediction accuracy for the 0K model is slightly smaller than that of the 1K-3K models, and the highest prediction accuracy of the 1K-3K models are similar. Similarly, the ROC curve (Figure 4b) and the precision-recall curve (Figure 4c) of the 0K model are slightly lower than the corresponding curves for the 1K-3K models. This result shows that the added information of degree correlations and transitivity do not influence the model enough to have a significant effect on the global prediction performance of functional homogeneity. Because the performance of the different *dK *distribution models are so similar, we hypothesized that the scores for the gene groups based on different *dK *models maybe highly correlated. We tested this hypothesis by studying the Spearman's correlation between the scores for the gene groups using different models. The results are given in Table 3. It can be seen that they are indeed highly correlated. Similar results were obtained based on the BIOGRID protein interaction network (Additional 2: Table S5).

**Table 3:** Spearman correlation between the scores of the gene groups for different *dK *distribution models based on MIPS protein interaction data.


**Spearman corrleation**	**0*K*-1*K***	**0*K*-2*K***	**0*K*-3*K***	**1*K*-2*K***	**1*K*-3*K***	**2*K*-3*K***

p = 0.9, gs = 10	0.9856	0.9833	0.9879	0.9994	0.9992	0.9992

p = 0.9, gs = 8	0.9767	0.9729	0.9787	0.9990	0.9989	0.9988

p = 0.85, gs = 10	0.9867	0.9839	0.9882	0.9994	0.9994	0.9993

p = 0.95, gs = 10	0.9835	0.9801	0.9851	0.9995	0.9996	0.9992

gs: group size

#### Results based on gene coexpression networks

**Figure 5:**
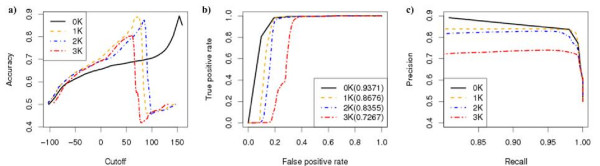
**The performance of *dK *distribution models in predicting functionally homogeneous modules based on GDS1013 coexpression data (PCC cut-off threshold 0.89)**. The gene group size was 10, the p-value cut-off threshold was 10^-5^, and *p *= 0.9. a) Accuracy; b) ROC curve; c) Precision-Recall. Note that the curve for the 0*K *model is truncated near (0.80,0.89); this is due to an abundance of modules having the maximum density, and no way to distinguish between them in the 0*K *measure. Therefore, the minimum recall computable is that shown in the graph.

We repeated the above analysis, this time using a yeast coexpression network built from a yeast microarray dataset, GDS1013, downloaded from the NCBI Gene Expression Omnibus [11]. We constructed the network by calculating the Pearson correlation coefficient (PCC) between the expression levels of every pair of genes. Then, each gene was represented as a node in the network, and we drew a link between two nodes if the PCC exceeded a certain threshold. To make the network as comparable as possible to the MIPS network, we selected the threshold such that the degree of the most highly connected node in each network was the same (286, which corresponded to a PCC cut-off threshold of 0.89). We were again surprised to find that the 2*K *performance was similar to 1*K*, while the 3*K *model's performance was actually worse than the 1*K *and 2*K *models (Figure 5). This observation maybe explained by the fact that the 3*K *model does not approximate the observed network well as shown in Table 2. Perhaps most strikingly, the 0*K *model displayed the best performance, showing that a simple measure of density is a very good predictor of function in coexpression networks. We also changed the threshold for the PCC between expression profiles to build the network to 0.93, the gene group size *n *= 8, and the parameter *p *to *p *= 0.85, 0.95 in defining the score function. The results are presented in Additional file 1: Figures S12-S18. Same qualitative results were obtained. We also studied the Spearman's correlation between the gene group scores for different *dK *models and the results are given in Table 4. Although the scores based on 1K, 2K, and 3K models are highly correlated, they do not strongly correlate with the scores based on the 0K model. Similar results were obtained for different PCC cut-off thresholds (Additional file 2: Table S6).

**Table 4:** Spearman correlation between the scores of the gene groups for different *dK *distribution models based on the GDS1013 coexpression network with PCC cut-off threshold of 0.89, *d *= 1, 2, 3.


**Spearman corrleation**	**0*K*-1*K***	**0*K*-2*K***	**0*K*-3*K***	**1*K*-2*K***	**1*K*-3*K***	**2*K*-3*K***

p = 0.9, gs = 10	0.2310	0.1759	0.0170	0.9489	0.8350	0.9247

p = 0.9, gs = 8	0.4419	0.4448	0.2920	0.9823	0.9073	0.9415

p = 0.85, gs = 10	0.3683	0.3491	0.1953	0.9606	0.8310	0.9149

p = 0.95, gs = 10	0.4433	0.4318	0.2637	0.9707	0.8490	0.9111

gs: group size

We also performed the same analysis for another gene expression dataset, GDS1103 [11]. The performance results for this dataset are presented in Additional file 1: Figures S19-S26 and Additional file 2: Tables S7-S8. It should be noted that none of the *dK *models performed well in identifying functionally homogeneous modules based on this gene expression data set. One potential reason is that the number of sampling points is only 11, which is much smaller than that of GDS1013, which has 24 sampling points. Thus the network constructed based on GDS1103 may not be reliable. Despite the drawbacks of this dataset, the conclusions from this dataset is qualitatively identical to those found for GDS1013. This demonstrates the generality of our conclusions with respect to gene coexpression networks.

## Conclusion

We studied the ability of *dK *distribution models to predict individual edges and functionally homogeneous modules in protein interaction and gene coexpression networks. A pseudo-likelihood logistic estimation method was proposed to estimate the parameters in the *dK *distribution models. We found that the 2*K *distribution model performs the best in predicting individual edges in both protein interaction and gene coexpression networks. A pseudo-likelihood ratio score function was then defined to evaluate potential functional homogeneity based on the *dK *distribution models. For yeast protein interaction networks, 1K, 2K and 3K models perform similarly and are slightly better than the 0K model in predicting functionally homogeneous modules. The *dK *scores were very highly correlated for different *d*. This means that, between two different subgraph topologies, the variation in the denominator, the *dK *distribution likelihood, was small relative to that in the numerator, the constant-*p *likelihood. In this case, most of the variation in scores between modules would be accounted for by the numerator. The different probabilities between 1*K*, 2*K*, and 3*K *may be similar overall in the networks we studied. For gene coexpression networks, the 0K model performs significantly better than the other models in predicting functionally homogeneous modules. We noted that 0*K*, or density, performed remarkably well as a prediction method even in the yeast protein interaction network, being able to find extremely functionally homogeneous groups of genes (*p *< 10^-5^). This may simply reflect that highly dense subnetworks in a protein interaction network represent protein complexes, which are of necessity functionally homogeneous.

One future avenue of research could be to remove this type of functionally homogeneous modules from the data, since they are relatively uninteresting examples of functional homogeneity. It may be that the subtle differences between the various *dK *distributions are useful to pick out homogeneous modules of more specific functions.

## Methods

### Data Sources

We downloaded yeast protein interaction data from two different data sources: MIPS [10] and BIOGRID [12]. The MIPS (Munich Information Center for Protein Sequences) dataset (version: PPI_18052006.tab) contains 12,319 protein physical interactions involving 4,546 proteins. The BIOGRID dataset (version 2.0.51) contains 91,364 protein physical interactions involving 5,563 proteins.

We also studied two gene expression datasets GDS1013 and GDS1103 downloaded from the NCBI Gene Expression Omnibus [11]. The GDS1013 expression data contains the expression profiles of about 6400 yeast genes and open reading frames by over-expressing the essential ribosomal protein activator IFH1. Twenty four samples of 2 growth protocols, 2 strains, and 5 time points were studied. Two genes are referred as linked if the Pearson correlation coefficient between the expression levels of every pair of genes is at least 0.89. The GDS1103 expression data studied the gene expression profiles of 6400 genes of leu3 mutant grown in either limited ethanol or limited ammonium media. Twelve samples involving 2 genotypes and 2 growth protocols were studied. To study the effect of different thresholds for coexpression in defining the networks, we used two threshold values 0.89 and 0.93 for the PCC between the gene expression profiles. In the main text, we provide our results based on the MIPS protein interaction data and the GDS1013 co-expression network with PCC cut-off threshold of 0.89. The results for the other networks are presented in the additional files.

### Model fitting for the *dK *models

The 0K model simply assigns a probability  to each edge independently. In the 1K model, edge occurrence is also independent of other edges, but only conditional on the degrees of their incident nodes. The probability is then given by

(1)

In this paper, we choose ϵ = 10^-4^.

In the 2K model, we parameterized as follows the probability

*p*(*k*_1_, *k*_2_) = *P *((*u, v*)|deg(*u*) = *k*_1_, deg(*v*) = *k*_2_), that two nodes of degrees *k*_1 _and *k*_2 _(*k*_1 _≤ *k*_2_) interact:

(2)

Based on the observed interaction network, we used logistic regression in Matlab to estimate the parameters (*α*, *β*, *γ*).

Finally, to fit the 3K model, we again reparameterized as follows. We classified each triplet of nodes according to their topology in the network. The topology definitions are shown in Figure 1, where the nodes 1, 2, 3 are in increasing order of degree (*k*_1 _≤ *k*_2 _≤ *k*_3_). We modeled the distribution of the triplet topology as a function of *Z *= (log *k*_1_, log *k*_2_, log *k*_3_).

(3)

where *p*_*i *_= *P*(topo(*X*) = *i*). We estimated the parameters by maximizing the pseudo-likelihood of the data. The pseudo-likelihood *Q *= *Q*(*α*_1_,⋯,*α*_7_; *β*_7_,⋯,*β*_7_) *α*_*i *_∈ *R*, *β*_*i *_∈ *R*^3^, 1 ≤ *i *≤ 7 is defined by multiplying the probability of the observed categories across all the triplets.

#### Calculate 2K distribution from 3K distribution

When we evaluate the ability of the 3K distribution model for predicting protein interactions in the next subsection, we need an equation linking the probability for two nodes to be connected based on the probabilities of the seven topologies given in Figure 1. The equation is given as follows.

Given degree pairs (*k*_1_, *k*_2_), with *k*_1 _≤ *k*_2_. We can get 2*K *distribution from 3*K *distribution as follows:

(4)

where .

### Evaluation of the *dK *models for predicting protein interactions

Based on the estimated parameters for the *dK *distribution models, the probability for each protein pair to interact can be predicted based on the above equations. Two proteins are referred as interacting if the predicted probability is above a cut-off threshold. We then compare the predicted interactions with the observed interactions. Since the number of non-interaction pairs is much larger than the number of interacting pairs, we randomly choose the same number of noninteracting pairs as that of the interacting pairs in this analysis. The comparisons between the observed and the predicted interactions are summarized as true positives (TP), true negatives (TN), false positives (FP), and false negatives (FN). The accuracy, true positive rate, false positive rate, precision, and recall are defined as







We studied the relationship between the accuracy and the cut-off threshold for the predicted probability of interactions, between the false positive rate and the true rate (the ROC curve), and between precision and recall.

### Random network simulation with *dK *distribution models

We generated 100 random networks based on the *dK *distribution models using the estimated parameters to see if the random networks approximate the observed network well. For each network, we calculate five statistics: the smallest eigenvalue of the Laplacian of the graph matrix, the largest eigenvalue of the Laplacian of the graph matrix, the shortest distance between the nodes, the standard deviation of shortest distance between the nodes, and the average assortativity coefficients as in [7]. These statistics give approximate description of the networks of interest. If the random networks approximate the true network well, these statistics should be close to the corresponding values of the true networks. The simulation steps were carried out as follows.

For the 1*K *distribution, we randomly rewired the edges of the observed network 50,000 times while preserving the degree distribution. To generate random instances of the 2*K *and 3*K *distributions, we used a simulated annealing approach to generate random networks. For the 2*K *model, the energy function is:

(5)

where *k*_max _is the maximum degree, *n*(*i*, *j*) the number of edges between pairs of nodes with degrees *i *and *j *in the observed network, *ñ*(*i*, *j*) is the predicted number of edges between pairs of nodes with degrees *i *and *j*, and (*i*, *j*) is the number of such edges in the randomized network. For the 3*K *model, the score function is:

(6)

where *n*_*l*_(*i*, *j*, *k*) is the number of occurrences of topology *l *(Figure 1) between triples of nodes with degrees *i*, *j *and *k *in the observed network, *ñ*_*l*_(*i, j, k*) is the predicted number of occurrences of topology *l *between triples of nodes with degrees *i*, *j *and *k*, and _*l*_(*i*, *j*, *k*) is the corresponding observed number in the randomized network. The detailed simulated annealing procedure is as follows.

Each state is a network. We find an initial state by rewiring the original network 10,000 times (preserving only the degree distribution). We then continue rewiring, but now we accept only resulting networks with lower energy scores, or with higher energy scores with probability , where *d *= 2 or 3, and *T *is the temperature, which we decrease as *T*_*k *_= *αT*_*k*-1_, with *T*_0 _= 1 and *α *= 0.995. We ran the simulated annealing for 50,000 iterations.

### Evaluation of model performance in identifying functionally homogeneous modules

We designed a pseudo-log-likelihood score function modeled after that used by Tanay et al. in the context of biclustering [8,9]. For a given module *M*, the denominator is the likelihood of the network topology in the subnetwork defined by the module, and the numerator is the likelihood under a high but constant probability *p *= 0.9 of each edge being present. For d = 0, 1, 2, the score is given by:

(7)

where *E *is the edge set of the network, and

(8)

where  is estimated by fitting the 2*K *model as described in equation (2)(*k*_1 _and *k*_2 _are the degrees of *u *and *v*.). For *d *= 3, we make use of the 8 cases defined in Figure 1, to which we refer as *t*_1_, *t*_2_,..., *t*_8_. The nodes 1, 2, and 3 in the figure are sorted by degrees. We denote topo(*u, v, w*) the topology of a node triplet (*u, v, w*), being one of *t*_1_,..., *t*_8_. (If one or more degrees are the same, some of the topologies will be interchangeable. We ignore this problem in the following formulation.)

(9)

Again,  is a function of the degrees of u, v, and w, and their topology *t*_*i*_, that we determined by fitting our model to the observed network.

Having defined a score function, we searched for modules of constant size and high score using a simulated annealing approach.

### Evaluation by functional homogeneity prediction

Given a set of gene modules (groups of genes) and their score in a network obtained by each of the four models, we measured model performance as follows. We tested the genes for enrichment in one or more functions in the "biological process" category of the Gene Ontology [13]. If the gene module showed a hypergeometric test *p*-value of less than 10^-5 ^(as we previously mentioned, the exact value is not critical to the results), we declared it "functionally homogeneous". This gave us True Positive and True Negative sets. We then tested how well a particular score could predict these categories by comparing Accuracy, ROC and Precision-Recall curves for each model.

### Simulated annealing search for high-scoring modules

We used the simulated annealing technique, described by Kirkpatrick [14], with the following definitions: A *state *is a subset of nodes from the network of fixed size *n*. The state space is therefore the set of all *n*-sized subsets of nodes from the full set of nodes of the network. The energy of a state is the negative of the pseudo-log-likelihood score described in equation (7) for 0K-2K models and equation (9) for the 3K model. A neighboring state is a subset that differs in exactly one member.

We ran the algorithm as follows:

• Set the initial temperature for the algorithm.

• Select a random set of *n *nodes, *S*_0_, to be the current state.

• While the temperature is less than a specified minimum temperature, perform the following steps:

- Select a putative next state by uniformly removing one node from the current module and uniformly adding a new one.

- Compute the energy of both the current state and the putative next state, *E*(*S*_*n*_) and *E*().

- Accept the next state with probability:



- Update the temperature: *T*_*n *_= *T*_0_/log(*n*).

## Authors' contributions

FS and YL provided the general ideas and guidance throughout the project. WW collected the data and estimated the parameters in the dK-models and JNI evaluated the models. WW and JNI drafted the manuscripts. FS and YL finalized the manuscript. All authors read and approve the final manuscript.

## Supplementary Material

Additional file 1 **Supplementary figures**. Interaction prediction results for BIOGRID, GDS1013 with PCC cut-off threshold 0.93 and GDS 1103; Performance of the *dK *distribution models for the identification of functional homogeneous modules for MIPS, BIOGRID, GDS1013 and GDS1103. Click here for file

Additional file 2 **Supplementary tables**. Comparison of five network features for the *dK *distribution models for BIOGRID, GDS1013 with PCC cut-off threshold 0.93 and GDS 1103; Spearman correlation between the scores of the gene groups for different dK distribution models based on BIOGRID, GDS1013 with PCC cut-off threshold 0.93 and GDS 1103. Click here for file
