# Supplementary material for: Usefulness and limitations of dK random graph models to predict interactions and functional homogeneity in biological networks under a pseudo-likelihood parameter estimation approach
Source: BMC Bioinformatics. 2009 Sep 3;10:277. doi: 10.1186/1471-2105-10-277 (PMC2755484; doi:10.1186/1471-2105-10-277)
Supplement: Additional file 1 — Supplementary figures. Interaction prediction results for BIOGRID, GDS1013 with PCC cut-off threshold 0.93 and GDS 1103; Performance of the dK distribution models for the identification of functional homogeneous modules for MIPS, BIOGRID, GDS1013 and GDS1103. [file 1471-2105-10-277-S1.pdf]

Supplementary Figures

Figure S1 - The performance of the dK distribution models in predicting protein interactions for the BIOGRID interaction data.

a) The prediction accuracy versus the cut-off threshold for the interaction probability; b) ROC curve; c) Precision-Recall.

Figure S2 - The performance of the dK distribution models in predicting protein interactions for the GDS1013 expression data (PCC cut-off threshold 0.93).

a) The prediction accuracy versus the cut-off threshold for the interaction probability; b) ROC curve; c) Precision-Recall.

Figure S3 - The performance of the dK distribution models in predicting protein interactions for the GDS1103 expression data (PCC cut-off threshold 0.89).

a) The prediction accuracy versus the cut-off threshold for the interaction probability; b) ROC curve; c) Precision-Recall.

Figure S4 - The performance of the dK distribution models in predicting protein interactions for the GDS1103 expression data (PCC cut-off threshold 0.93).

a) The prediction accuracy versus the cut-off threshold for the interaction probability; b) ROC curve; c) Precision-Recall.

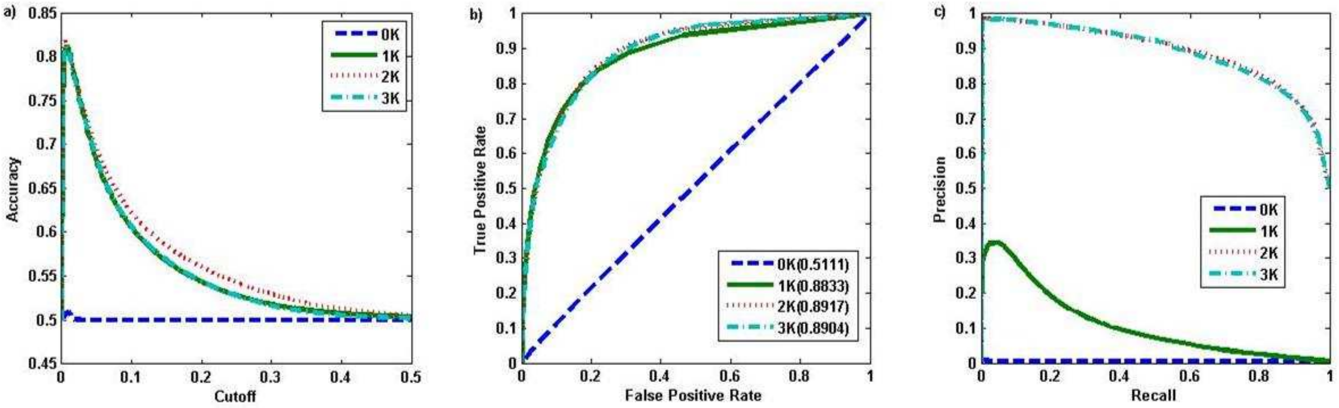

Figure S1

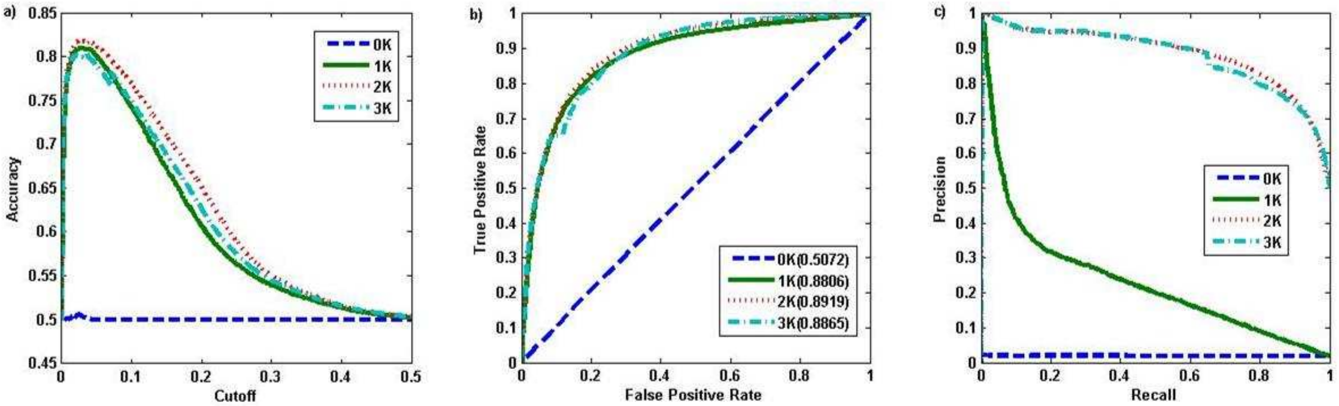

Figure S2

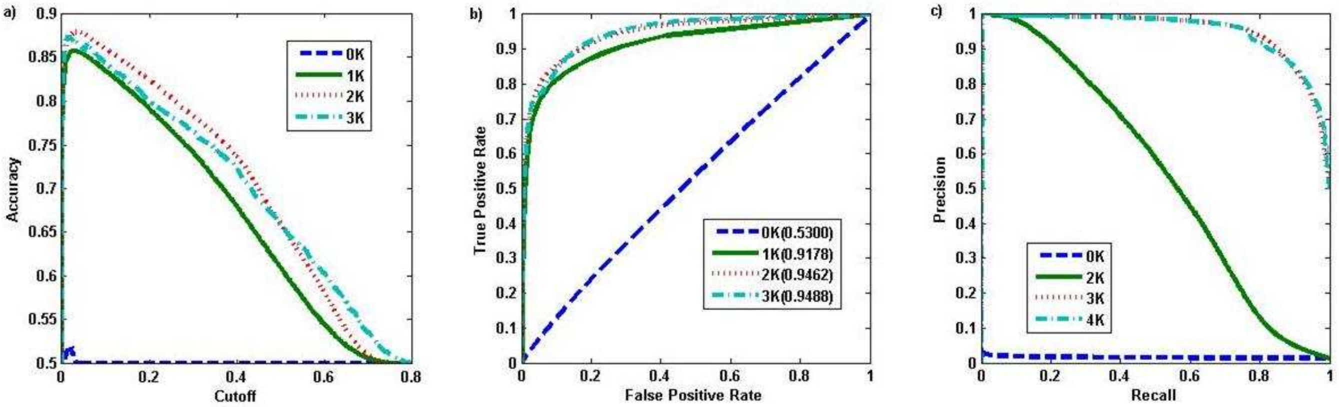

Figure S3

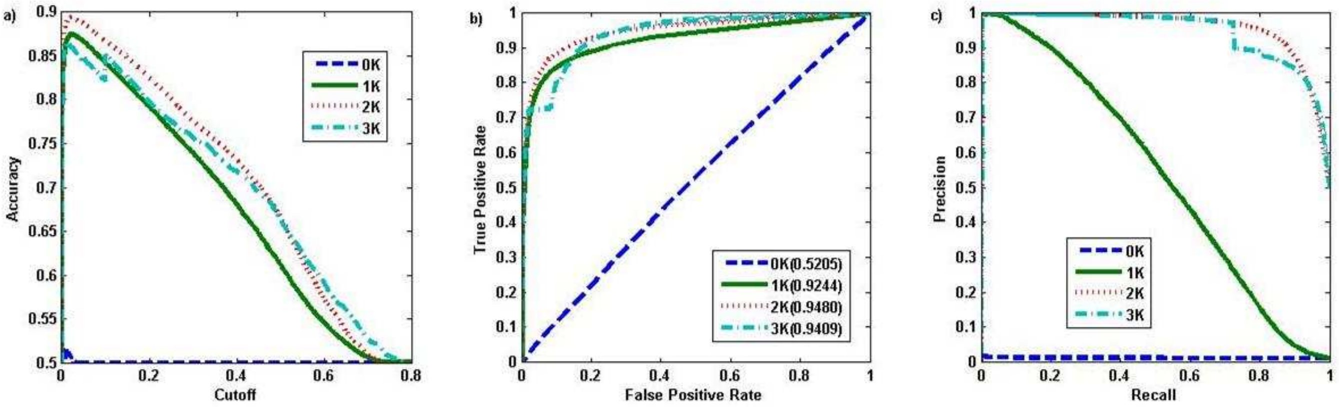
a)
Accuracy
Cutoff
0K
1K
2K
3K
b)
True Positive Rate
False Positive Rate
0K(0.5205)
1K(0.9244)
2K(0.9480)
3K(0.9409)
c)
Precision
Recall
0K
1K
2K
3K

Figure S4

Figure S5 - The performance of dK distribution models in predicting function homogeneous modules based on MIPS interaction data.

The gene group size was 8, the p-value cut-off was 10^{-5}, and $p = 0.9$. a) Accuracy; b) ROC curve; c) Precision-Recall.

Figure S6 - The performance of dK distribution models in predicting function homogeneous modules based on MIPS interaction data.

The gene group size was 10, the p-value cut-off was 10^{-5}, and $p = 0.85$. a) Accuracy; b) ROC curve; c) Precision-Recall.

Figure S7 - The performance of dK distribution models in predicting function homogeneous modules based on MIPS interaction data.

The gene group size was 10, the p-value cut-off was 10^{-5}, and $p = 0.95$. a) Accuracy; b) ROC curve; c) Precision-Recall.

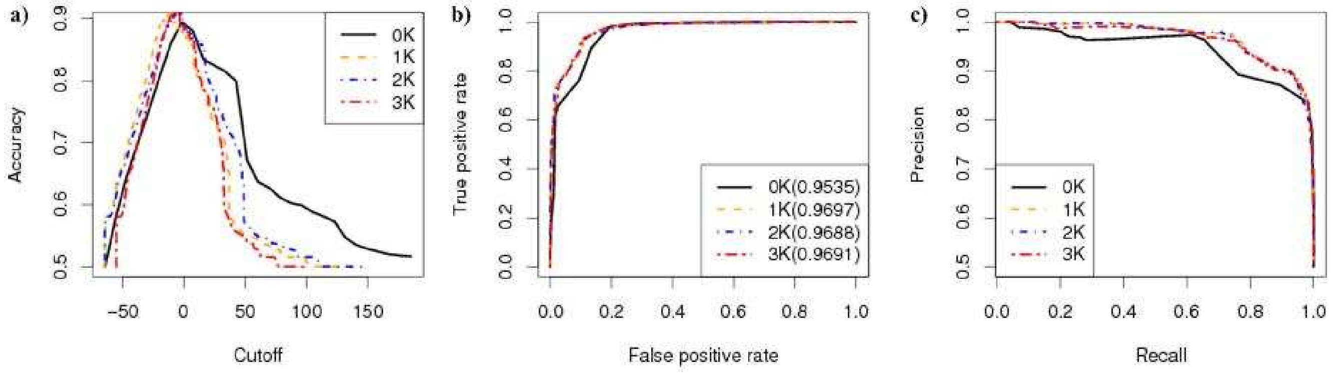

Figure S5

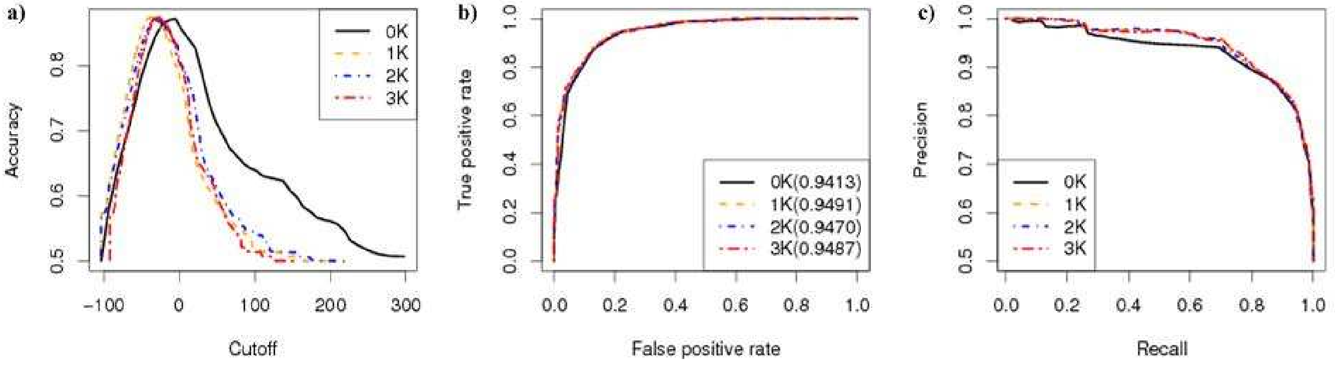

Figure S6

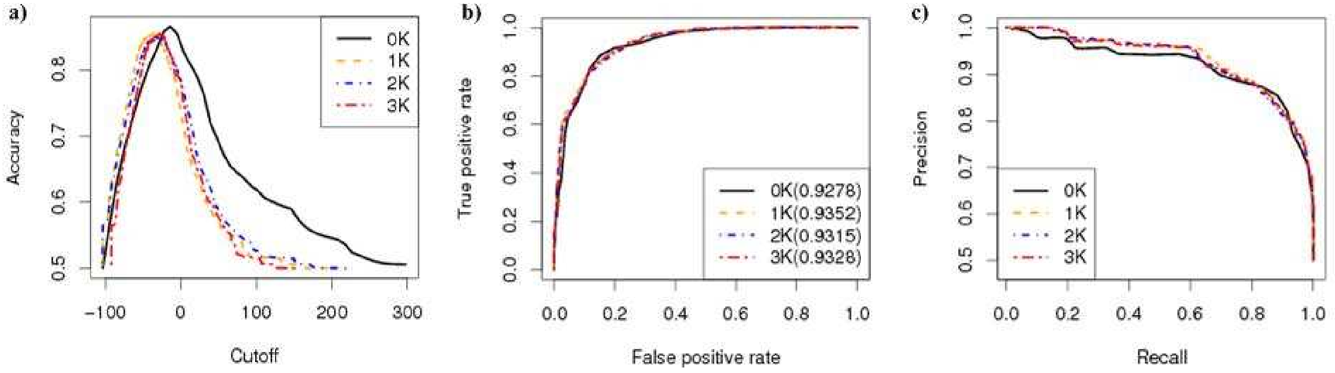

Figure S7

Figure S8 - The performance of dK distribution models in predicting function homogeneous modules based on BIOGRID interaction data.

The gene group size was 8, the p-value cut-off was 10^{-5}, and $p = 0.9$. a) Accuracy; b) ROC curve; c) Precision-Recall.

Figure S9 - The performance of dK distribution models in predicting function homogeneous modules based on BIOGRID interaction data.

The gene group size was 10, the p-value cut-off was 10^{-5}, and $p = 0.9$. a) Accuracy; b) ROC curve; c) Precision-Recall.

Figure S10 - The performance of dK distribution models in predicting function homogeneous modules based on BIOGRID interaction data.

The gene group size was 10, the p-value cut-off was 10^{-5}, and $p = 0.85$. a) Accuracy; b) ROC curve; c) Precision-Recall.

Figure S11 - The performance of dK distribution models in predicting function homogeneous modules based on BIOGRID interaction data.

The gene group size was 10, the p-value cut-off was 10^{-5}, and $p = 0.95$. a) Accuracy; b) ROC curve; c) Precision-Recall.

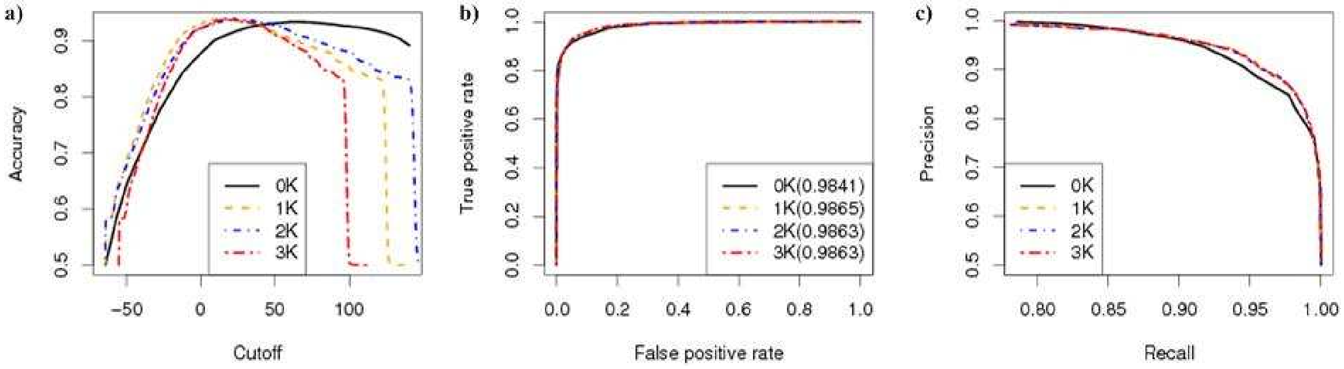

Figure S8

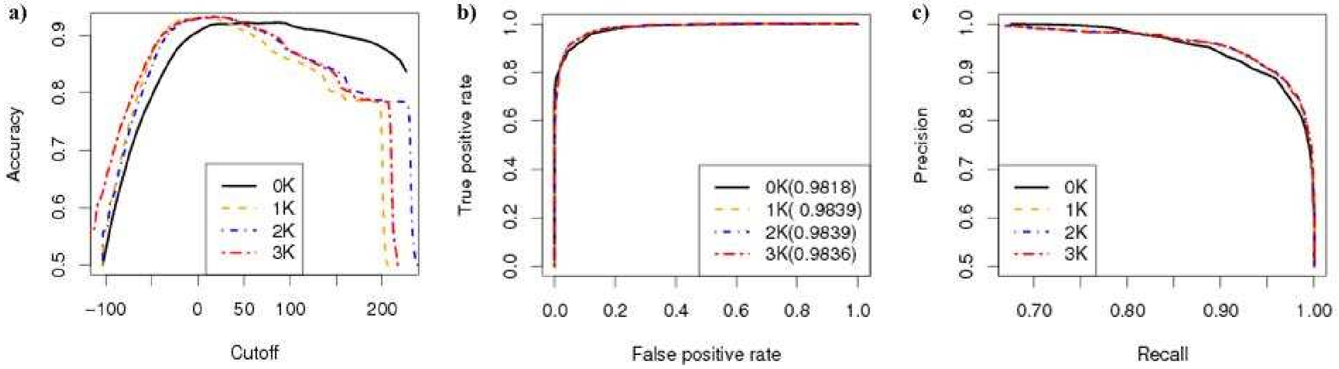

Figure S9

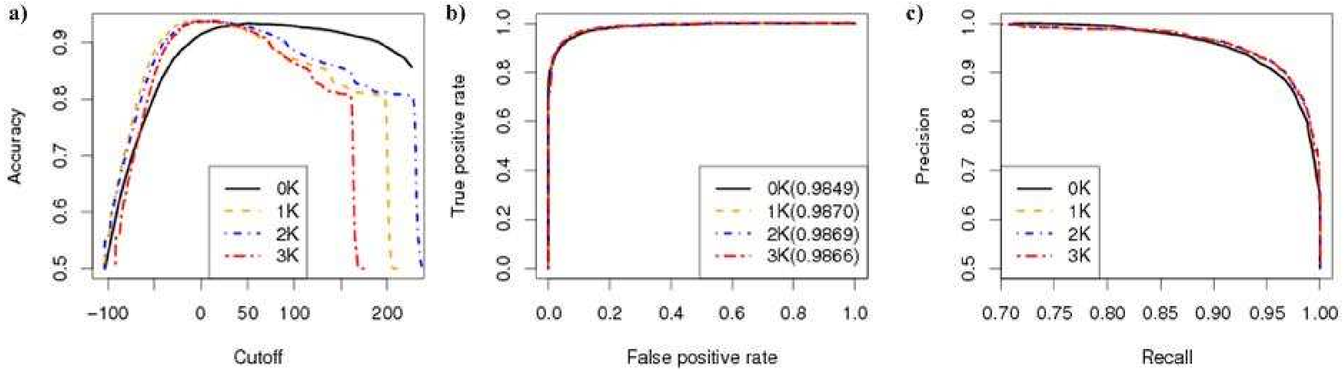

Figure S10

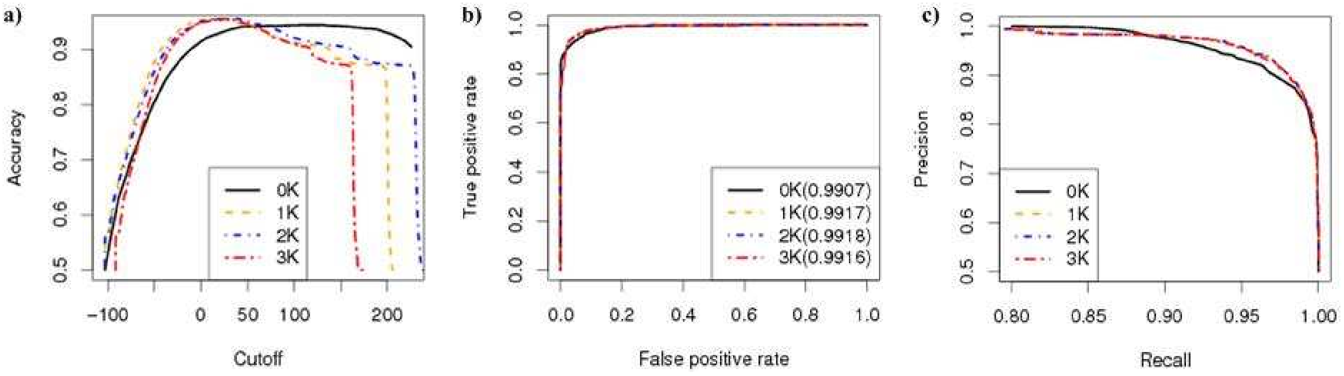

Figure S11

Figure S12 - The performance of dK distribution models in predicting function homogeneous modules based on GDS1013 coexpression data (PCC cut-off threshold 0.89).

The gene group size was 8, the p-value cut-off was 10^{-5}, and $p = 0.9$. a) Accuracy; b) ROC curve; c) Precision-Recall.

Figure S13 - The performance of dK distribution models in predicting function homogeneous modules based on GDS1013 coexpression data (PCC cut-off threshold 0.89).

The gene group size was 10, the p-value cut-off was 10^{-5}, and $p = 0.85$. a) Accuracy; b) ROC curve; c) Precision-Recall.

Figure S14 - The performance of dK distribution models in predicting function homogeneous modules based on GDS1013 coexpression data (PCC cut-off threshold 0.89).

The gene group size was 10, the p-value cut-off was 10^{-5}, and $p = 0.95$. a) Accuracy; b) ROC curve; c) Precision-Recall.

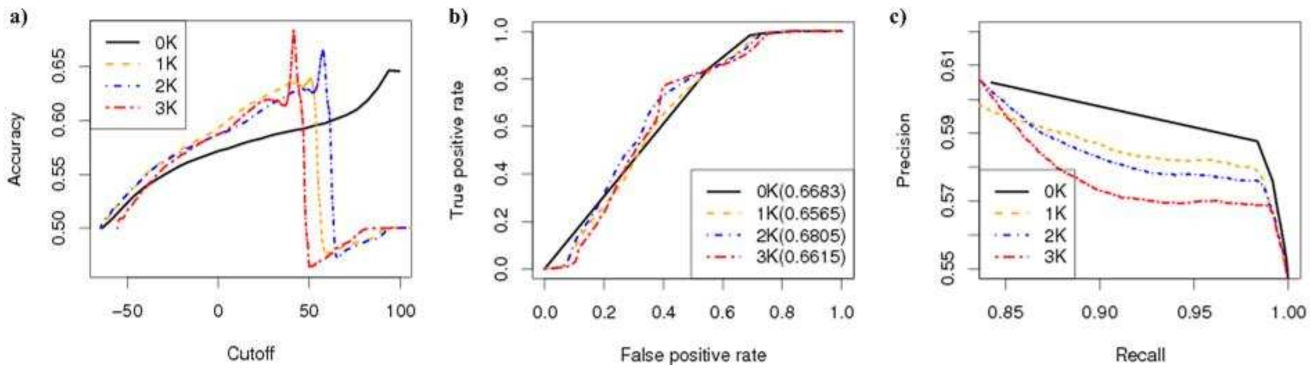

Figure S12

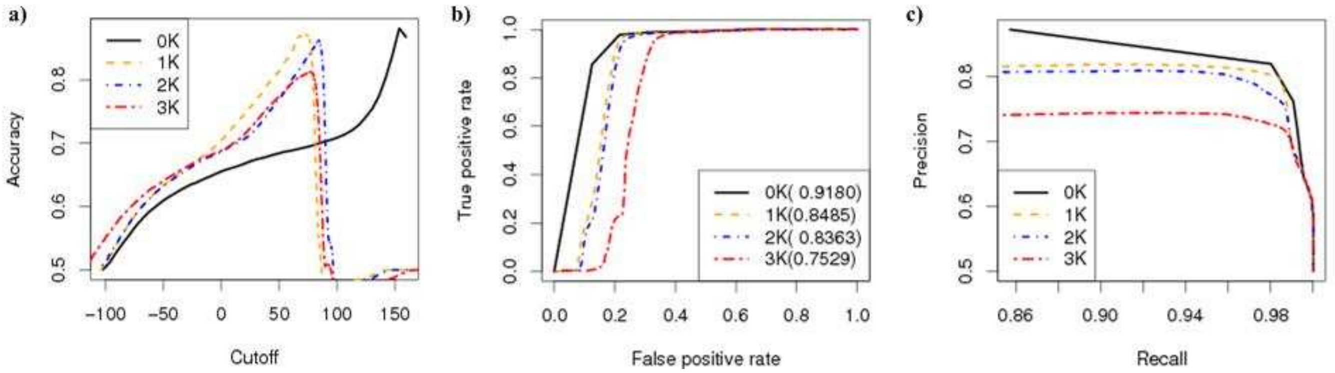

Figure S13

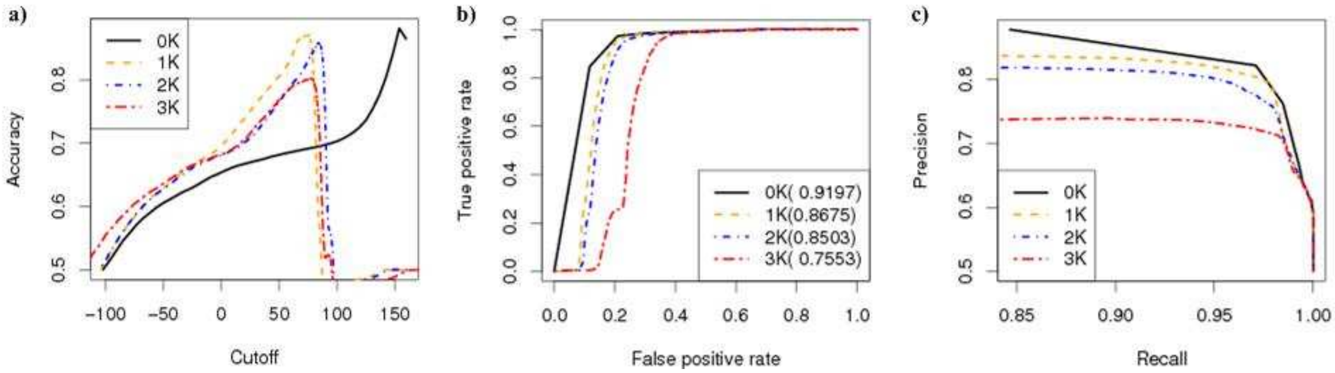

Figure S14

Figure S15 - The performance of dK distribution models in predicting function homogeneous modules based on GDS1013 coexpression data (PCC cut-off threshold 0.93).

The gene group size was 8, the p-value cut-off was 10^{-5}, and $p = 0.9$. a) Accuracy; b) ROC curve; c) Precision-Recall.

Figure S16 - The performance of dK distribution models in predicting function homogeneous modules based on GDS1013 coexpression data (PCC cut-off threshold 0.93).

The gene group size was 10, the p-value cut-off was 10^{-5}, and $p = 0.9$. a) Accuracy; b) ROC curve; c) Precision-Recall.

Figure S17 - The performance of dK distribution models in predicting function homogeneous modules based on GDS1013 coexpression data (PCC cut-off threshold 0.93).

The gene group size was 10, the p-value cut-off was 10^{-5}, and $p = 0.85$. a) Accuracy; b) ROC curve; c) Precision-Recall.

Figure S18 - The performance of dK distribution models in predicting function homogeneous modules based on GDS1013 coexpression data (PCC cut-off threshold 0.93).

The gene group size was 10, the p-value cut-off was 10^{-5}, and $p = 0.95$. a) Accuracy; b) ROC curve; c) Precision-Recall.

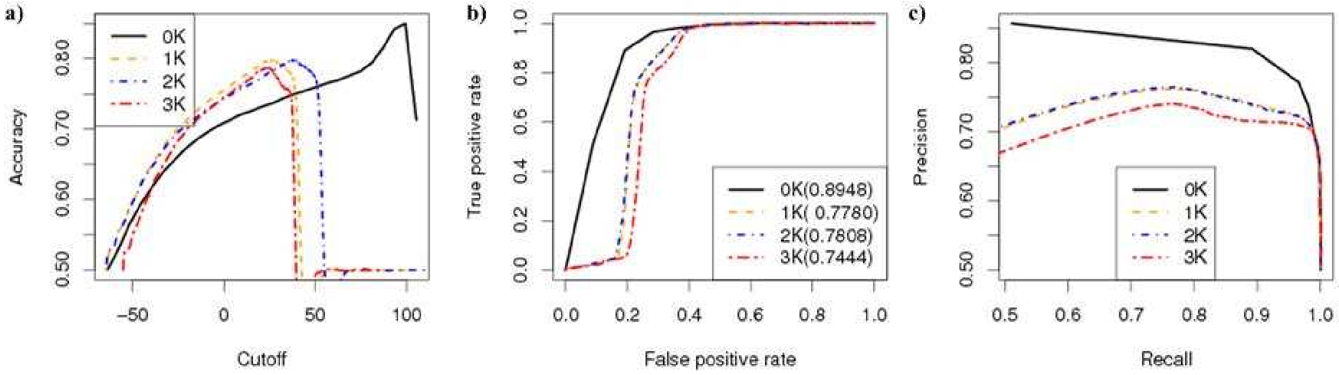

Figure S15

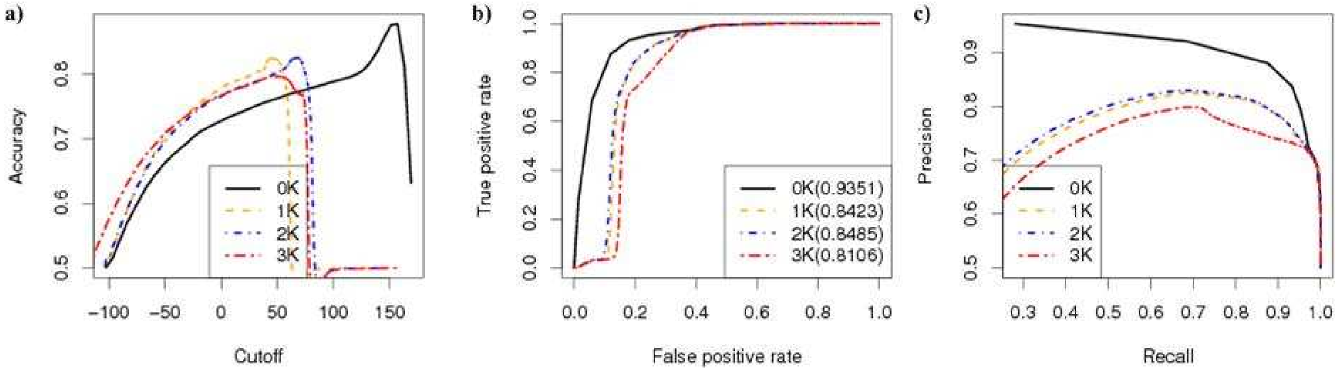

Figure S16

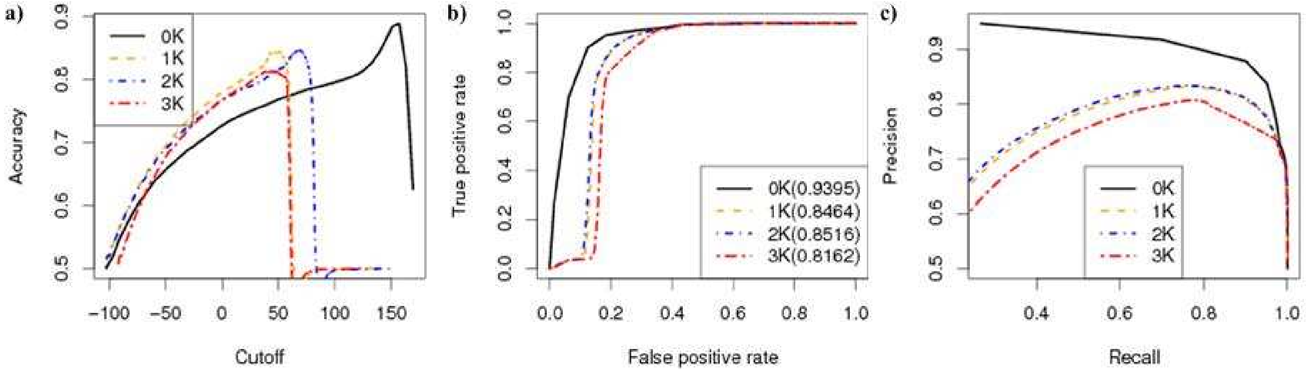

Figure S17

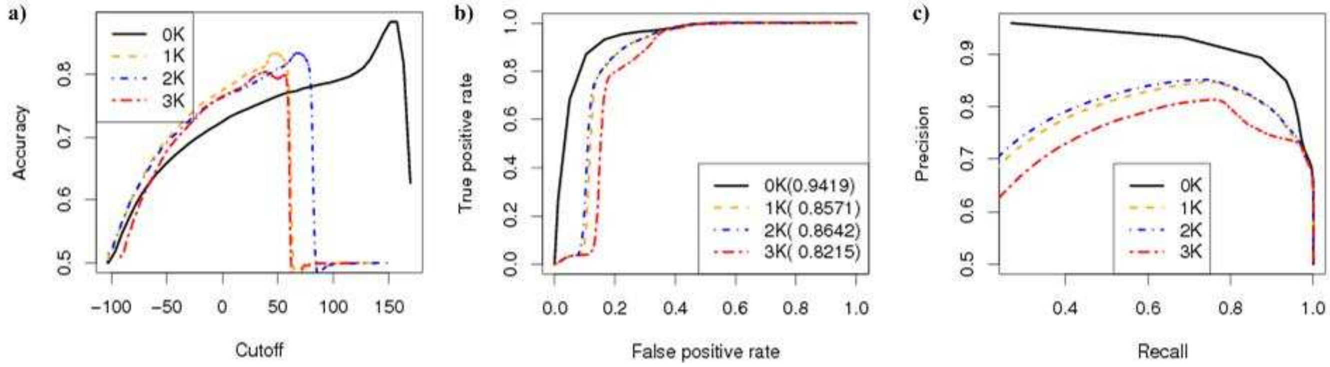

Figure S18

Figure S19 - The performance of dK distribution models in predicting function homogeneous modules based on GDS1103 coexpression data (PCC cut-off threshold 0.89).

The gene group size was 8, the p-value cut-off was 10^{-5}, and $p = 0.9$. a) Accuracy; b) ROC curve; c) Precision-Recall.

Figure S20 - The performance of dK distribution models in predicting function homogeneous modules based on GDS1103 coexpression data (PCC cut-off threshold 0.89).

The gene group size was 10, the p-value cut-off was 10^{-5}, and $p = 0.9$. a) Accuracy; b) ROC curve; c) Precision-Recall.

Figure S21 - The performance of dK distribution models in predicting function homogeneous modules based on GDS1103 coexpression data (PCC cut-off threshold 0.89).

The gene group size was 10, the p-value cut-off was 10^{-5}, and $p = 0.85$. a) Accuracy; b) ROC curve; c) Precision-Recall.

Figure S22 - The performance of dK distribution models in predicting function homogeneous modules based on GDS1103 coexpression data (PCC cut-off threshold 0.89).

The gene group size was 10, the p-value cut-off was 10^{-5}, and $p = 0.95$. a) Accuracy; b) ROC curve; c) Precision-Recall.

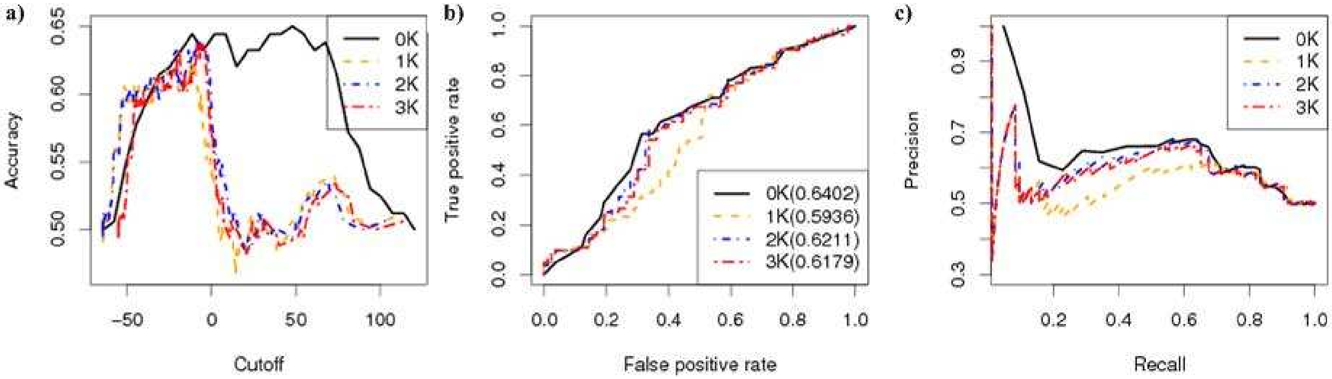

Figure S19

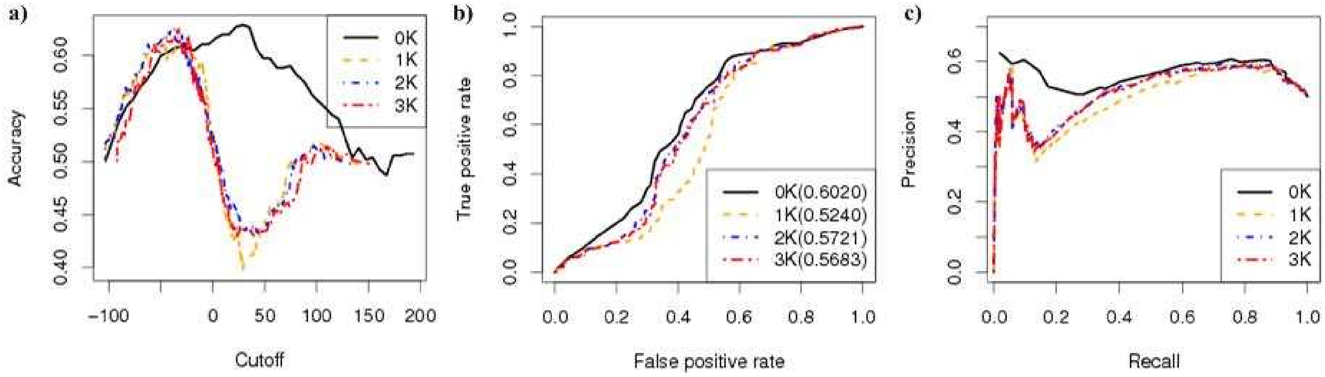

Figure S20

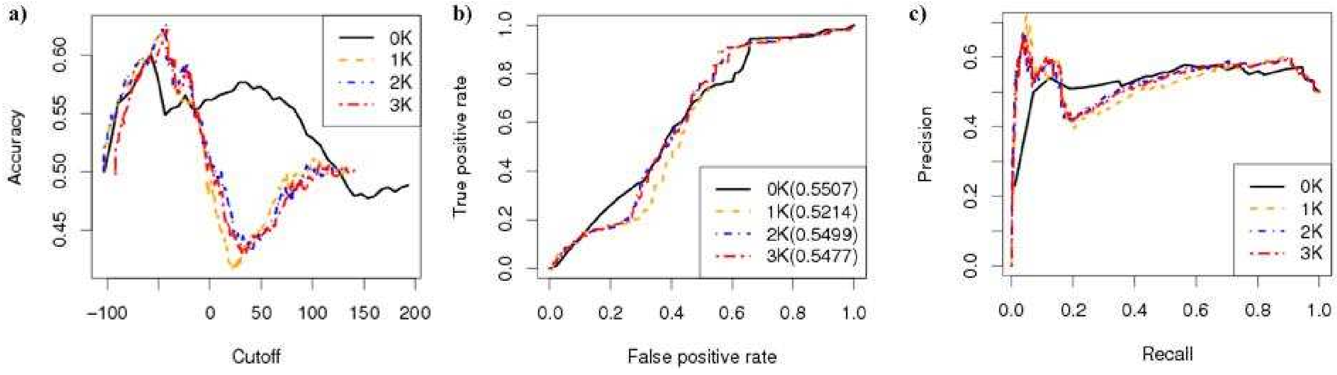

Figure S21

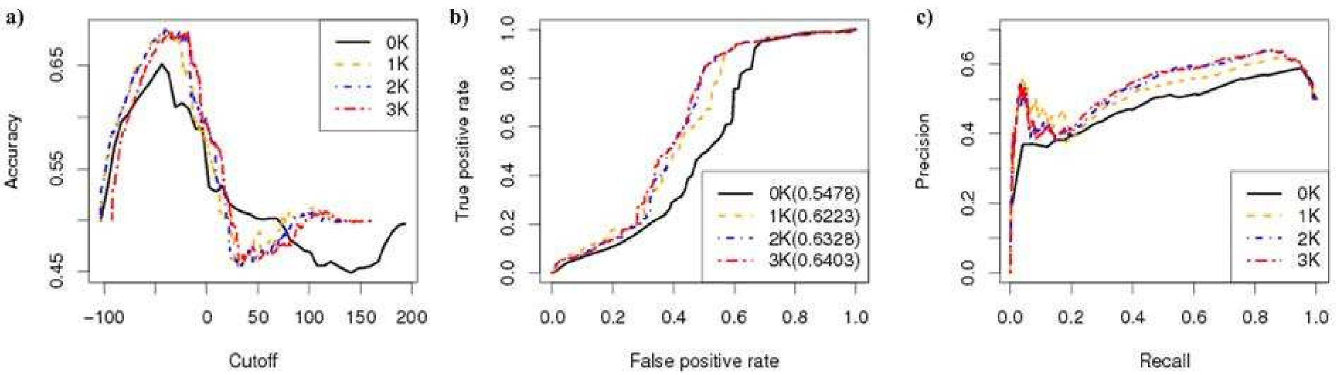
a)
Accuracy
Cutoff
0K
1K
2K
3K
b)
True positive rate
False positive rate
0K(0.5478)
1K(0.6223)
2K(0.6328)
3K(0.6403)
c)
Precision
Recall
0K
1K
2K
3K

Figure S22

Figure S23 - The performance of dK distribution models in predicting function homogeneous modules based on GDS1103 coexpression data (PCC cut-off threshold 0.93).

The gene group size was 8, the p-value cut-off was 10^{-5}, and $p = 0.9$. a) Accuracy; b) ROC curve; c) Precision-Recall.

Figure S24 - The performance of dK distribution models in predicting function homogeneous modules based on GDS1103 coexpression data (PCC cut-off threshold 0.93).

The gene group size was 10, the p-value cut-off was 10^{-5}, and $p = 0.9$. a) Accuracy; b) ROC curve; c) Precision-Recall.

Figure S25 - The performance of dK distribution models in predicting function homogeneous modules based on GDS1103 coexpression data (PCC cut-off threshold 0.93).

The gene group size was 10, the p-value cut-off was 10^{-5}, and $p = 0.85$. a) Accuracy; b) ROC curve; c) Precision-Recall.

Figure S26 - The performance of dK distribution models in predicting function homogeneous modules based on GDS1103 coexpression data (PCC cut-off threshold 0.93).

The gene group size was 10, the p-value cut-off was 10^{-5}, and $p = 0.95$. a) Accuracy; b) ROC curve; c) Precision-Recall.

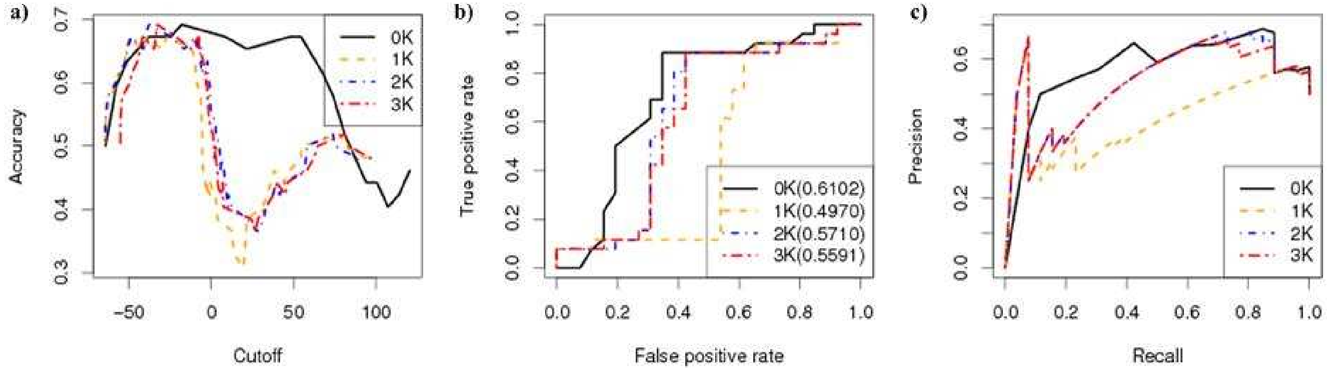

Figure S23

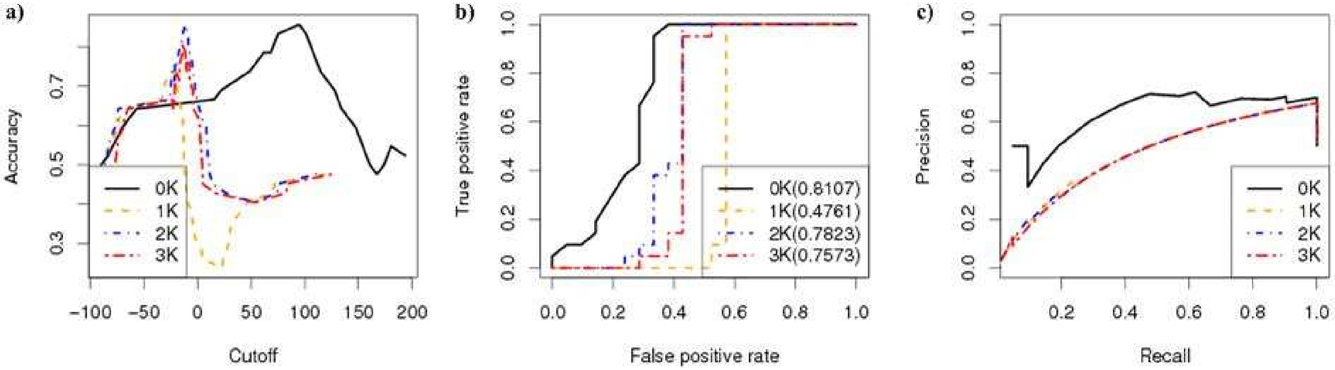

Figure S24

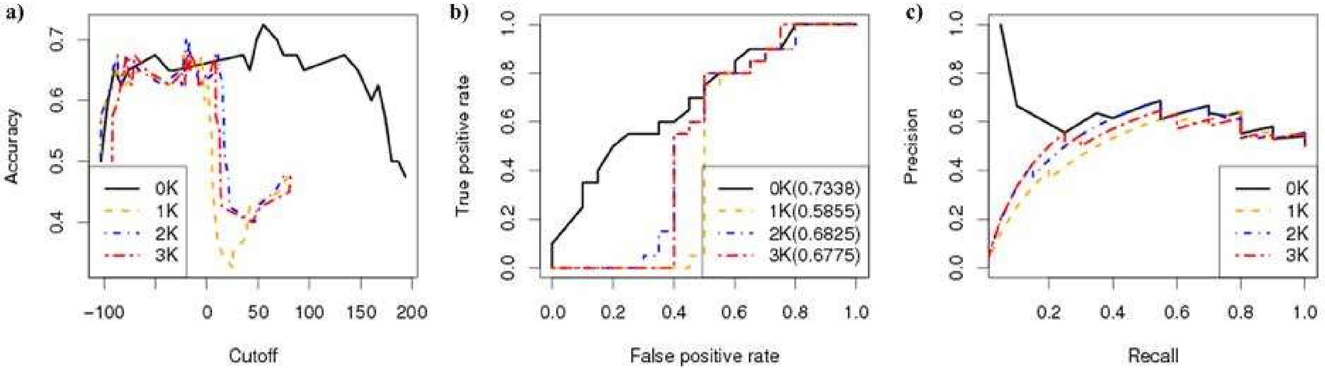

Figure S25

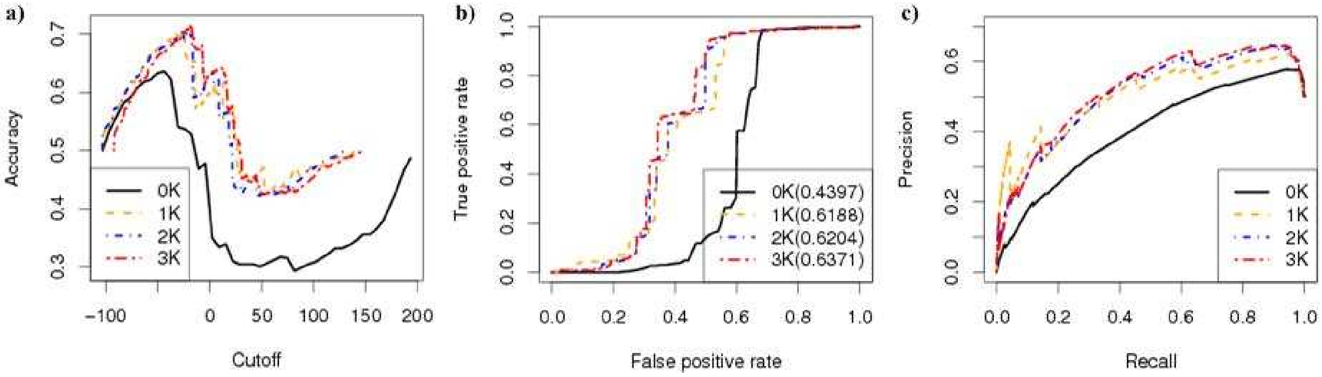

a)
Accuracy
Cutoff
0K
1K
2K
3K
b)
True positive rate
False positive rate
0K(0.4397)
1K(0.6188)
2K(0.6204)
3K(0.6371)
c)
Precision
Recall
0K
1K
2K
3K

Figure S26
