# Supplementary material for: Usefulness and limitations of dK random graph models to predict interactions and functional homogeneity in biological networks under a pseudo-likelihood parameter estimation approach
Source: BMC Bioinformatics. 2009 Sep 3;10:277. doi: 10.1186/1471-2105-10-277 (PMC2755484; doi:10.1186/1471-2105-10-277)
Supplement: Additional file 2 — Supplementary tables. Comparison of five network features for the dK distribution models for BIOGRID, GDS1013 with PCC cut-off threshold 0.93 and GDS 1103; Spearman correlation between the scores of the gene groups for different dK distribution models based on BIOGRID, GDS1013 with PCC cut-off threshold 0.93 and GDS 1103. [file 1471-2105-10-277-S2.pdf]

Supplementary Tables

Table S1 - Comparison of five network features for the dK distribution models with that of the BIOGRID protein interaction network, $d = 1, 2, 3$.

Metric	λ_1	λ_{n-1}	d	σ_d	r
Biogrid	0.23	1.77	2.84	0.64	-0.09
1k	0.28(0.030)	1.72(0.030)	2.79(0.0025)	0.61	-0.07(0.0012)
2k	0.25(0.028)	1.75(0.028)	2.80(0.0021)	0.62	-0.083(0.0010)
3k	0.24(0.026)	1.76(0.026)	2.81(0.0022)	0.62	-0.088(0.0009)

λ_1: average of the smallest eigenvalue of the Laplacian of the graph matrix; λ_{n-1}: average of the largest eigenvalue of the Laplacian of the graph matrix; d: average shortest distance between the nodes; σ_d: standard deviation of shortest distance between the nodes; r: average assortativity coefficients.

Table S2 - Comparison of five network features for the dK distribution models with that of the coexpression network from GDS1013 with PCC cut-off threshold 0.93, $d = 1, 2, 3$.

Metric	λ_1	λ_{n-1}	d	σ_d	r
Coexp	0.06	1.94	3.98	1.46	0.24
1k	0.31(0.070)	1.69(0.070)	2.78(0.0073)	0.72	-0.05(0.0082)
2k	0.18(0.047)	1.82(0.047)	2.93(0.0015)	0.84	0.13(0.0054)
3k	0.24(0.055)	1.76(0.055)	2.87(0.0089)	0.78	0.12(0.0043)

Notations are the same as in Table S1.

Table S3 - Comparison of five network features for the dK distribution models with that of the coexpression network from GDS1103 with PCC cut-off threshold 0.89, $d = 1, 2, 3$.

Metric	λ_1	λ_{n-1}	d	σ_d	r
Coexp	0.05	1.95	4.87	1.84	0.49
1k	0.17(0.054)	1.83(0.054)	2.76(0.0058)	0.67	0.02(0.0028)
2k	0.07(0.020)	1.93(0.020)	3.13(0.0018)	0.97	0.35(0.0017)
3k	0.06(0.021)	1.94(0.021)	3.15(0.0017)	1.12	0.36(0.0018)

Notations are the same as in Table S1.

Table S4 - Comparison of five network features for the dK distribution models with that of the coexpression network from GDS1103 with PCC cut-off threshold 0.93, $d = 1, 2, 3$.

Metric	λ_1	λ_{n-1}	d	σ_d	r
Coexp	0.02	1.98	6.05	2.95	0.46
1k	0.26(0.039)	1.74(0.039)	2.86(0.0059)	0.65	-0.12(0.0046)
2k	0.25(0.045)	1.75(0.045)	2.87(0.0060)	0.66	-0.13(0.0060)
3k	0.15(0.044)	1.85(0.044)	2.97(0.0087)	0.72	0.13(0.0037)

Notations are the same as in Table S1.

Table S5 - Spearman correlation between the scores of the gene groups for different dK distribution models based on BIOGRID protein interaction data.

Spearman correlation	$0K$-$1K$	$0K$-$2K$	$0K$-$3K$	$1K$-$2K$	$1K$-$3K$	$2K$-$3K$
p = 0.9, gs = 8	0.9603	0.9600	0.9601	0.9996	0.9957	0.9975
p = 0.9, gs = 10	0.9672	0.9668	0.9669	0.9997	0.9973	0.9985
p=0.85, gs = 10	0.9614	0.9611	0.9610	0.9996	0.9963	0.9980
p=0.95, gs = 10	0.9116	0.9113	0.9106	0.9991	0.9895	0.9944

gs: group size

Table S6 - Spearman correlation between the scores of the gene groups for different dK distribution models based on the GDS1013 coexpression network with PCC cut-off threshold of 0.93, $d = 1, 2, 3$.

Spearman correlation	$0K$-$1K$	$0K$-$2K$	$0K$-$3K$	$1K$-$2K$	$1K$-$3K$	$2K$-$3K$
p = 0.9, gs = 8	0.8109	0.8331	0.7597	0.9946	0.9833	0.9780
p = 0.9, gs = 10	0.7046	0.7528	0.6151	0.9859	0.9688	0.9497
p=0.85, gs = 10	0.7203	0.7712	0.6350	0.9837	0.9650	0.9428
p=0.95, gs = 10	0.7856	0.8272	0.7054	0.9869	0.9674	0.9501

gs: group size

Table S7 - Spearman correlation between the scores of the gene groups for different dK distribution models based on the GDS1103 coexpression network with PCC cut-off threshold of 0.89, $d = 1, 2, 3$.

Spearman correlation	$0K$-$1K$	$0K$-$2K$	$0K$-$3K$	$1K$-$2K$	$1K$-$3K$	$2K$-$3K$
p = 0.9, gs = 8	0.7140	0.8531	0.6321	0.9227	0.9567	0.9654
p = 0.9, gs = 10	0.6723	0.5632	0.5572	0.8931	0.9089	0.9691
p=0.85, gs = 10	0.7447	0.5548	0.5436	0.9321	0.9402	0.9786
p=0.95, gs = 10	0.6169	0.5544	0.6453	0.9233	0.9413	0.9763

gs: group size

Table S8 - Spearman correlation between the scores of the gene groups for different dK distribution models based on the GDS1103 coexpression network with PCC cut-off threshold of 0.93, $d = 1, 2, 3$.

Spearman correlation	$0K$-$1K$	$0K$-$2K$	$0K$-$3K$	$1K$-$2K$	$1K$-$3K$	$2K$-$3K$
p = 0.9, gs = 8	0.9170	0.9631	0.9569	0.9573	0.9582	0.9991
p = 0.9, gs = 10	0.8727	0.9639	0.9573	0.9221	0.9239	0.9991
p=0.85, gs = 10	0.8987	0.9558	0.9496	0.9563	0.9576	0.9993
p=0.95, gs = 10	0.9169	0.9544	0.9473	0.9715	0.9723	0.9993

gs: group size
